# Measuring the placebo effect in carpal tunnel syndrome

**DOI:** 10.1186/s10195-019-0540-4

**Published:** 2020-01-28

**Authors:** Jordi Faig-Martí, Adriana Martínez-Catassús

**Affiliations:** Hospital Sant Rafael, Pg. Vall d’Hebron 107-117, 08035 Barcelona, Catalonia Spain

**Keywords:** Placebo effect, Randomized trial, Expectations, Doctor–patient communication, Carpal tunnel syndrome

## Abstract

**Background:**

The placebo effect can account for part of the improvement seen in patients undergoing any type of treatment, be it surgical or pharmacological. The objective of this study is to quantify the placebo effect in carpal tunnel syndrome treatment.

**Materials and methods:**

A double-blinded randomized trial was performed with 68 patients suffering from mild to moderate carpal tunnel syndrome, divided into two groups with no statistically significant differences regarding age, weight, or degree of nerve compression. The patients were evaluated clinically and electromyographically before and after 2 months of treatment with either palmitoylethanolamide (PEA) or placebo.

**Results:**

The results, comparing the two groups, showed an improvement in both groups on a visual analogue scale (VAS) and Levine’s questionnaire, which have been reported to show statistical differences in only a few items. In the placebo group, the mean age was 53.32 years (±13.43) and the BMI was 28.85 kg/m^2^ (±4.84). Before treatment, the average symptom severity score (SSS) on the Levine questionnaire was 2.57 (±0.74) and the functional status score (FSS) was 2.24 (±0.66). After treatment, these decreased to 2.11 (±0.81) and 1.96 (±0.77), being statistically nonsignificant for SSS (*p* = 0.0865) but significant for FSS (*p* = 0.0028). VAS showed a statistically nonsignificant decrease from 4.06 to 3.25 (*p* = 0.3407). After placebo treatment, SSS, FSS, and VAS improved by 0.46, 0.28, and 0.81 points or 17.89%, 12.5%, and 19.95%, respectively.

**Conclusions:**

These results show an improvement in the studied parameters by up to 20%, but when compared with those published in literature, these show great variability due to the wide variety of factors involved in the placebo effect. Several factors that affect the placebo effect are discussed, and the present work tries to quantify it in carpal tunnel syndrome.

**Level of Evidence:**

Level 2 of evidence according to “The Oxford 2011 Level of Evidence.”

## Introduction

A placebo is defined as an inert or nonactive treatment, that is, a substance or treatment of no intended therapeutic value [1]. The term comes from the Latin verb “to please.” Common placebos include inert tablets such as sugar pills, inert injections such as saline, sham therapies, or sham surgery [2].

Placebos are usually employed in clinical trials to control for nonspecific effects but have been found to cause clinically relevant changes and, in some cases, be indistinguishable from the treatment against which they are tested [3]. They are used to help researchers to determine the effectiveness of a drug or treatment. In this way, treatments that are usually considered effective and even used as standard of care can be challenged [4]. The first placebo-controlled trial is believed to have been conducted in 1799 [5]. When a person who is taking the inactive substance or who has had a sham treatment reports that symptoms have improved, this improvement is called the placebo effect.

There are multiple possible components of a measured placebo effect. Some of them depend on the patient, such as the past effect of active treatments, physiological mediators (expectations, desires, and emotions), brain structures involved in reward/aversion and regulation of emotions, endogenous opioids or endocannabinoids [6], or classical conditioning. Other factors influencing the placebo effect depend on the physician, such as empathy or cues that signal that an active treatment has been given, or depend on the type of treatment.

A significant amount of research has been carried out in the field of pain and analgesia, and the placebo analgesic response appears to be the best-understood model of placebo mechanisms. However, other placebo responses result from less conscious processes, such as classical conditioning in the case of immune, hormonal, and respiratory functions [5].

Various published studies comparing placebo or sham therapies with treatments in neuropathic pain, especially in carpal tunnel syndrome (CTS), are available. Some recent studies compared neurodynamic techniques, platelet-rich plasma ultrasound-guided injection, low-level laser therapy, minocycline, topical chamomile oil, radial shock wave therapy, oscillatory biofield therapy, acetyl-l-carnitine (ALCAR), gabapentin, and topical menthol with placebo or sham therapies for treatment of mild and moderate CTS.

Giving a placebo to a patient when there is an effective treatment available is a bioethically complex issue. Informed consent is usually required for a study to be considered ethical, including the disclosure that some test subjects will receive placebo treatment. The ethics of placebo-controlled studies were debated in the revision process of the Declaration of Helsinki [5].

The aim of the present study is to draw attention to the placebo effect and quantify it in the conservative treatment of CTS.

## Materials and methods

A double-blinded randomized study was conducted with 68 patients with a diagnosis of mild to moderate CTS divided into two groups receiving either palmitoylethanolamide (PEA) or placebo during 60 days. The study was approved by the ethics committee of our institution. Exclusion criteria were history of previous upper extremity surgery, active treatment with steroids, use of night splinting, or food allergies. Patients selected for the study had to be between 18 and 75 years of age and suffer from mild or moderate CTS for at least 3 months with an electromyographical study (EMG) to confirm the diagnosis. Patients were asked to sign an informed consent form to be included in this study. Before and 2 months after treatment, a clinical and electromyographical study (EMG) were conducted, registering pain on a visual analogue scale (VAS), presence of Durkan, Tinel, and Phalen’s signs, and the Levine questionnaire. Levine’s questionnaire, which includes a symptom severity score (SSS) and a functional score (FSS), was conducted in a Spanish translation before and after the treatment.

Group N included the patients receiving 600 mg PEA, while patients in group P received placebo pills that looked exactly the same for 60 days. Thereafter, another EMG and clinical study including the Levine questionnaire were performed, recording any complications that the patient might refer. Two patients (one in each group) did not complete the treatment for causes unrelated to the medication, and five more did not have a follow-up EMG due to refusal or loss of the report. The 61 patients with full reports included 18 females and 12 males in group N, and 19 females and 12 males in group P. The right hand was affected in 39 patients, and the left one in 25. Fibromyalgia was diagnosed in three patients in group N but not in group P. The results regarding treatment with PEA have already been published [7], and the present report focuses on the placebo effect in the treatment of mild and moderate CTS.

Data were analyzed using the R project statistical package [8] with a *p*-value of > 0.05. The *t*-test was used for data following a normal distribution, and the Wilcoxon rank-sum test with continuity correction was used for quantitative data. Presence or absence of clinical signs were assessed statistically using the chi-squared test. The *t*-test was used to search for statistically significant changes in the values of VAS, Levine’s questionnaire, and EMG.

## Results

The average age in group P was 53.32 years (SD 13.43) with an average weight of 72.43 kg (SD 13.03) and BMI of 28.85 kg/m^2^ (SD 4.84). No complications due to the use of placebo were recorded. The recorded values of the studied parameters are presented in Table 1. Note that, in this table, all subjective parameters improved, although only SSS and the total Levine score reached a statistically significant level.

**Table 1:** Average parameters before and after treatment (SD in brackets) with their variation

	Pretreatment	Posttreatment	Difference	*p*-Value	*t*-Value
VAS	4.06 (3.42)	3.25 (3.18)	−0.8 (3.08)	0.1554	1.4572
FSS	2.24 (0.66)	1.96 (0.77)	−0.27 (0.87)	0.08656	1.772
SSS	2.57 (0.74)	2.11 (0.81)	−0.45 (0.78)	0.002809*	3.255
Levine questionnaire	2.409	2.04	−0.368	0.01251*	2.657
Sensitive speed	40.53 (7.05)	42 (8.54)	2.13 (9.08)	0.262	−1.15
Sensitive peak amplitude	8.86 (5.42)	11.12 (6.13)	1.198 (5.22)	0.2625	−1.1476
Motor latency	3.87 (0.91)	3.71 (0.7)	−0.057 (0.41)	0.5345	0.632

*Statistically significant data

## Discussion

The magnitude of the placebo effect is very difficult to assess due to the many factors involved (Table 2). Studies that quantify the placebo effect show very different values depending on the condition treated and each of the items being measured. In low back pain, for example, the reduction of pain measured by VAS showed a reduction that ranged from 29% for maximum pain to 46% for minimum pain, while back pain-related disability decreased by 40% and pain bothersomeness decreased by 34% [9]. In a metaanalysis on knee osteoarthritis, placebo treatment produced a 22–27% VAS reduction and 28–29% improvement in WOMAC score [10]; and in a CTS study, placebo showed a reduction in night discomfort of 7.7%, swelling of 11.5%, movement discomfort of 13%, and poor coordination of 5.2% [11]. In a study of CTS treated with neurodynamic techniques, the placebo group showed a 2.9% reduction in VAS. Amanzio [12] studied the placebo effect versus analgesics and showed differences in pain intensity of 1–1.5 out of 10 units in a VAS attributable to the placebo effect. These improvements in pain intensity can reach 5 units out of 10 in patients considered as placebo responders [13].

**Table 2:** Factors involved in the placebo effect

Patient and environment or psychosocial determinants of the placebo effect	Pathology involved Measured item Past effect of active treatments Psychological mediators (expectations, desires, and emotions) Brain structures involved in reward/aversion and regulation of emotions Endogenous opioids Classical conditioning
Physician	Cues that signal that an active treatment has been given Empathy
Type of treatment	Type of administration Look of the product

In the present study, the measured improvement of CTS with placebo in patient-dependent items ranged from 12.05% for FSS and 17.51% for SSS to 19.7% for VAS; And in objective items, such as those measured by EMG, the improvement was 1.47% for motor latency, 5.26% for sensitive speed, and 13.52% for sensitive peak amplitude. These results imply a spontaneous improvement of CTS in our patients during the period of study, which would add to the improvement due to the placebo effect.

Psychological factors that influence the placebo effect include classical conditioning, past experiences of the patient, especially those involving the use of active treatments, but also expectations, desires, and emotions.

The placebo effect also has a neuroanatomic basis that has been proven in magnetic resonance imaging (MRI) studies. Endogenous opioids have also shown to be involved in the placebo effect. Levine showed in 1978 [14] that analgesia provided by placebo could be antagonized by naloxone, indicating mediation by endogenous opioid systems. Furthermore, several neurobiologists have shown that the placebo effect reduces neural activity in brain areas related to anxiety and pain and increases that in areas involved in emotional regulation [15]. Thus, placebo produces a real biological effect that would not preclude its use in medical practice on the grounds of ethical reasons. However, we are aware that these issues can be difficult to deal with in the clinical setting, especially when explaining our treatment plan to the patient and their family. Telling the patient and their family that we are using a placebo is bound to make it disappear, but on the other hand, it would be unethical to give the patient a medication about which they are not informed. Curiously enough, this vision is not supported by an article by Kaptchuk et al. [16] that showed persistence of the placebo effect in spite of the patient knowing about it. This is a highly controversial topic, but many physicians use placebo in their practice [17].

Physicians’ attitudes towards the patient and their communicating skills also influence the placebo effect, with both active treatments and placebo. The way the physician communicates can sometimes give inadvertent cues that, in a clinical trial, the product prescribed is the active one: not only by the act of prescription, but also through a global attitude that can instill confidence, empathy, and reassurance, and so to say, prepare the brain for pain control [18]. Conditioning, verbal suggestions, and behaviors manifested by healthcare providers can determine placebo responses, as demonstrated in studies that used open instead of hidden administration of a drug, for example, using a computer-programmed drug infusion pump [12, 19–21].

The type of treatment used also has an effect on the final result. Thus, it is well known that more aggressive treatments are seen as more effective [22], so intravenous medication will be seen as more effective than oral medication by the patient [23]. Even the color of the administered substance contributes to the placebo effect; Also, when surgery is an option, it is also seen as more effective than conservative treatment [4].

One of the weaknesses of this study is that the results cannot be split between a group of placebo responders and another of nonresponders. The results should therefore be interpreted as those from a general population.

In the present study, pain measured by VAS should rather be considered a measure of bothersomeness, since the main problem in the condition studied is paresthesia and not pain itself.

As we have seen, several factors influence the placebo effect. Subtle differences in these factors can have a huge impact on the measured response to a treatment, which could explain the variability in the measured outcomes of placebo studies. Therefore, the relative contribution of each of these factors remains to be established. In the clinical setting, care providers can ethically take advantage of this placebo effect to enhance the effectiveness of treatments with proven results [24].

## Data Availability

The datasets used and/or analyzed during the current study are available from the corresponding author on reasonable request.

## References

[1] Arnstein P, Broglio K, Wuhrman E, Kean MB (2011). Use of placebos in pain management. Pain Manag Nurs.

[2] Lanotte M, Lopiano L, Torre E, Bergamasco B, Colloca L, Benedetti F (2005). Expectation enhances autonomic responses to stimulation of the human subthalamic limbic region. Brain Behav Inmunity.

[3] Gaab J (2019). The placebo and its effects: a psychoneuroendocrinological perspective. Psychoneuroendocrinology.

[4] Beard DJ, Rees JL, Cook JA, Rombach I, Cooper C, Merritt N, Shirkey BA, Donovan JL, Gwilym S, Savulescu J, Moser J, Gray A, Jepson M, Tracey I, Judge A, Wartolowska K, Carr AJ (2018). Arthroscopic subacromial decompression for subacromial shoulder pain (CSAW): a multicentre, pragmatic, parallel group, placebo-controlled, three-group, randomised surgical trial. Lancet.

[5] Price DD, Finniss DG, Benedetti F (2008). A comprehensive review of the placebo effect: recent advances and current thought. Annu Rev Psychol.

[6] Corcoran L, Roche M, Finn DP (2015). The role of the brain’s endocannabinoid system in pain and its modulation by stress. Int Rev Neurobiol.

[7] Faig-Martí J, Martínez-Catassús A (2017). Use of palmitoylethanolamide in carpal tunnel syndrome: a prospective randomized study. J Orthop Traumatol.

[8] R_Development_Core_Team (2011). R: a language and environment for statistical computing.

[9] Carvalho C, Caetano JM, Cunha L, Rebouta P, Kaptchuk TJ, Kirsch I (2016). Open-label placebo treatment in chronic low back pain: a randomized controlled trial. Pain.

[10] Saltzman BM, Leroux T, Meyer MA, Basques BA, Chahal J, Bach BR, Yanke AB, Cole BJ (2017). The therapeutic effect of intra-articular normal saline injections for knee osteoarthritis: a meta-analysis of evidence level 1 studies. Am J Sports Med.

[11] Spooner GR, Desai HB, Angel JF, Reeder BA, Donat JR (1993). Using pyridoxine to treat carpal tunnel syndrome. Randomized control trial. Can Fam Physician.

[12] Amanzio M, Pollo A, Maggi G, Benedetti F (2011). Response variability to analgesics: a role for non-specific activation of endogenous opioids. Pain.

[13] Benedetti F (1996). The opposite effects of the opiate antagonist naloxone and the cholecystokinin antagonist proglumide on placebo analgesia. Pain.

[14] Levine JD, Gordon NC, Fields HL (1978). The mechanism of placebo analgesia. Lancet.

[15] Wager T, Rilling JK, Smith EE, Sokolik A, Casey KL (2004). Placebo-induced changes in fMRI in the anticipation and experience of pain. Science.

[16] Kaptchuk TJ, Friedlander E, Kelley JM, Sanchez MN, Kokkotou E, Singer JP, Kowalczykowski M, Miller FG, Kirsch I, Lembo AJ (2010). Placebos without deception: a randomized controlled trial in irritable bowel syndrome. PLoS ONE.

[17] Tilburt JC, Emanuel EJ, Kaptchuk TJ, Curlin FA, Miller FG (2008). Prescribing “placebo treatments”: results of National Survey of U.S. internists and rheumatologists. Br Med J.

[18] Kaptchuk TJ, Kelley JM, Conboy LA, Davis RB, Kerr CE, Jacobson EE, Kirsch I, Schyner RN, Nam BH, Nguyen LT, Park M, Rivers AL, McManus C, Kokkotou E, Drossman DA, Goldman P, Lembo AJ (2008). Components of placebo effect: randomised controlled trial in patients with irritable bowel syndrome. BMJ.

[19] Benedetti F, Pollo A, Lopiano L, Lanotte M, Vighetti S (2003). Conscious expectation and unconscious conditioning in analgesic; motor and hormonal placebo/nocebo responses. J Neurosci.

[20] Levine JD, Gordon NC (1984). Influence of the method of drug administration on analgesic response. Nature.

[21] Colloca L, Lopiano L, Lanotte M, Benedetti F (2004). Overt versus covert treatment for pain, anxiety, and Parkinson’s disease. Lancet Neurol.

[22] Doherty M, Dieppe P (2009). The “placebo” response in osteoarthritis and its implications for clinical practice. Osteoarthr Cartil.

[23] Zhang W, Robertson J, Jones AC, Dieppe PA, Doherty M (2008). The placebo effect and its determinants in osteoarthritis: meta-analysis of randomised controlled trials. Ann Rheum Dis.

[24] Bystad M, Bystad C, Wynn R (2015). How can placebo effects best be applied in clinical practice? A narrative review. Psychol Res Behav Manag.

